# Supplementary figures and images for: The amino acid composition of a protein influences its expression
Source: PLoS One. 2024 Oct 14;19(10):e0284234. doi: 10.1371/journal.pone.0284234 (PMC11472945; doi:10.1371/journal.pone.0284234)

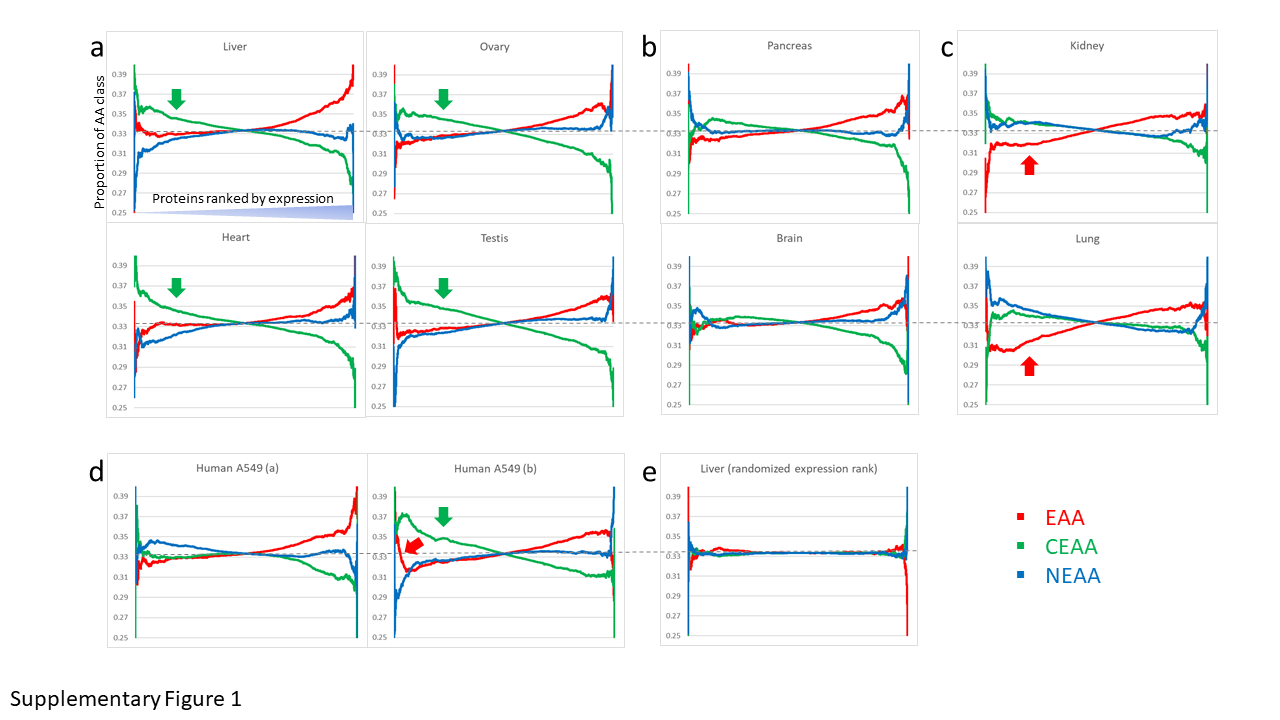

Supplement: S1 Fig — A smoothing procedure (see Methods and S1 File) was applied to visualise trends in relative, ranked amino acid class proportion when plotted against ranked protein expression level for 8 human tissues and two samples of a lung cancer cell line, A549 (data from PaxDB). As described in the main text, tissues can be placed in three groups (a, b, and c) based on the profile of EAA/CEAA/NEAA composition across the range of expression levels. A549 differences (d) most likely represent amino acid constraint effects brought about by different proliferation rates. Graph e shows the same liver data as in a but with randomised expression level as a control. (TIF) [file pone.0284234.s001.tif]
